# The Calcineurin Inhibitor FK506 Prevents Cognitive Impairment by Inhibiting Reactive Astrogliosis in Pilocarpine-Induced Status Epilepticus Rats

**DOI:** 10.3389/fncel.2017.00428

**Published:** 2018-01-09

**Authors:** Jinzhi Liu, Zhihua Si, Shuqing Li, Zhan Huang, Yan He, Tao Zhang, Aihua Wang

**Affiliations:** Department of Neurology, Shandong Provincial Qianfoshan Hospital, Shandong University, Shandong, China

**Keywords:** epilepsy, cognitive impairment, astrogliosis, neuroprotection, FK506

## Abstract

Status epilepticus (SE) is a severe clinical manifestation of epilepsy accompanying with cognitive impairment and brain damage. Astrocyte activation occurs following seizures and plays an important role in epilepsy-induced pathological injury, including cognitive impairment. FK506, an immunosuppressant used in clinical settings to prevent allograft rejection, has been shown to exhibit neuroprotective effects in central nervous system diseases. The present study was designed to investigate the effect of FK506 on cognitive impairment in a lithium-pilocarpine-induced SE rat model. It's found that FK506 treatment significantly increased the latency period to seizures and decreased the maximal intensity of seizures. FK506 treatment also markedly increased the surviving cells and reduced the neuron apoptosis after seizures. Meanwhile, FK506 treatment reduced the escape latency and prolonged the swimming distance in the Morris water maze test. In addition, FK506 treatment down-regulated the expression level of GFAP, a specific marker of astrocytes. In conclusion, FK506 could prevent and recover cognitive impairment by inhibiting reactive astrogliosis in pilocarpine-induced status epilepticus rats, suggesting that FK506 may be a promising agent for the treatment of epilepsy.

## Introduction

Status epilepticus (SE) refers to a seizure that lasts for over 30 min or repeated seizures, without returning to the normal state (Motamedi and Meador, 2003). Epilepsy is a paroxysmal neurological disorder, which is accompanied by a number of physiological and neurochemical changes, such as cognitive impairment, astrocyte activation, and neuronal necrosis (Motamedi and Meador, 2003; Rodriguez-Alvarez et al., 2015). It is reported that multiple interacting factors may affect cognitive function of epileptic patients (Motamedi and Meador, 2003). Hippocampus, which plays a key role in learning and memory, is the main region that neurological alterations occur (Rodriguez-Alvarez et al., 2015). Previous research demonstrated that neuronal loss and marked gliosis were associated with hippocampal sclerosis in patients with mesial temporal lobe epilepsy (MTLE) (Byeon et al., 2015). Pyramidal neuronal loss and degeneration are common in various regions of the hippocampus in a range of animal models of epilepsy (Elsharkawy et al., 2009; Pestana et al., 2010). Changes in the hippocampus, including reactive gliosis, are reported to be the cause of epilepsy-induced cognitive impairment (Swann, 2004; Jansson et al., 2009).

Astrocytes are the most common type of glial cells in the central nervous system. Besides providing trophic support for neurons, astrocytes sense brain microenvironment and interact with neurons, thus regulating neuronal function, plasticity, and signaling (de Lanerolle et al., 2010). They are thought to be of importance for the homeostasis of neurons (Ricci et al., 2009). In epileptic seizures, astrocytes are related to the heightened neuronal excitability and abnormal synchronization of discharge in the neuronal network (Agarwal et al., 2011). Astrogliosis is an abnormal increase in the number of astrocytes due to the destruction of nearby neurons from disease including neurodegenerative disease (Ricci et al., 2009; Peixoto-Santos et al., 2015). Astrogliosis is pathological hallmarks of the epileptic brain and thought to contribute to seizure generation in epilepsy (Ricci et al., 2009; Peixoto-Santos et al., 2015).

The calcineurin inhibitor FK506 is an effective immunosuppressant used in the clinic to prevent the allograft rejection (Malvezzi and Rostaing, 2015; Zou et al., 2015). It exerts its immunosuppressive action by binding to FK506 binding protein 12 (Wang et al., 2014; Vervliet et al., 2015). Recent studies demonstrated that FK506 played a role in both neuroprotection and neurotrophy (Kaminska et al., 2004; Nito et al., 2011). Despite the neuronal protective effect of FK506 observed in experimental cerebral ischemia and seizure models, the mechanism by which FK506 targets neurons remains unclear. It was reported that FK506 may protect neurons by preventing the sprouting of mossy fibers in the hippocampus in kainate-induced seizures (Gant et al., 2015).

In our present study, a lithium pilocarpine-induced SE rat model was used to examine the effect of FK506 on cognitive impairment after SE and its role in the modulation of reactive astrogliosis.

## Materials and methods

### Experimental animals

Adult male Wistar rats weighing 250–280 g were purchased from Experimental Animal Center of Shandong University, China. The rats were housed under controlled temperature and light conditions (12 h light; 12 h dark cycle, with lights on at 08:00 a.m.), with free access to food and water. The experimental procedures were approved by the ethics committee of Shandong University and performed in accordance with international standards for experiments on animals.

### Pretreatment of animals

The rats were randomly divided into three groups (15 rats in each group, *n* = 45): the control group, the pilocarpine-treated group, and the FK506-treated group. The rats in the control group were injected with a single administration of 0.9% saline, intraperitoneally. The rats in the pilocarpine-treated group received a single administration of 0.9% pilocarpine, intraperitoneally. The rats in the FK506-treated group received an intraperitoneal injection of 2 mg/kg FK506 (Fujisawa, Japan) 24 h prior to the administration of pilocarpine, and another injection of 2 mg/kg FK506 were given 1 h before the administration of pilocarpine. The dose of pilocarpine in the FK506-treated group was the same as that in the pilocarpine-treated group.

### Induction of seizures

To prevent peripheral cholinergic effects on the central nervous system, lithium chloride (3 mEq/kg, intraperitoneal), which does not cross the blood–brain barrier, was administered 24 h prior to subcutaneous injection (s.c.) of 1 mg/kg scopolamine methyl nitrate, followed by the treatment of pilocarpine for 30 min. All efforts were made to reduce the number of animals used and minimize animal suffering. After 1 h, the rats developed convulsive seizures at stage 4 or 5 according to Racine as follows (Racine, 1972): stage 0, no spasm indicated; stage 1, facial myoclonus represented; stage 2, head nodding suggested; stage 3, forelimb clonus represented; stage 4, rearing along with severe forelimb clonus indicated; stage 5, rearing and falling together with severe forelimb clonus. SE was terminated by diazepam (10 mg/kg, intraperitoneal).

### Morris water maze test

After the induction of SE, the experimental animals put on black belts and were trained in a Morris water maze every day for 7 days to assess their spatial learning and memory abilities. The water maze consisted of a tank filling with opaque powdered milk (0.5–1.5%, 22–25°C). The tank with the diameter of 1.2 m and the height of 0.5 m was separated into four parts (marked as A–D) in the form of clockwise. A hidden rigid platform (12 cm in diameter) was placed 1 cm below the surface of the water in quadrant D. An automated video tracking system (Ethovision 2.0; Noldus, Wageningen, Netherlands) was used for synchronous tracking of the animals' behavior. The Morris water maze tests included place navigation tests, which examine the ability of the rats to perform a specific location-based task, and spatial probe tests, which examine their ability to retain spatial memory (Pouzet et al., 2002). In the place navigation tests, the rats took part in four trials every day for 5 consecutive days. The escape latency (time from start point to reach the hidden platform) was used to assess the learning and memory performance of the rats. The platform is in quadrant D of the tank. The rats were placed in a determined start point and the longest escape latency was 120 s on each trial. The trial was terminated if the rats found a platform or the escape latency is over 120 s. If the rats failed to find the hidden platform within 120 s, they were allowed on the platform for a short time. In the experiment, we mainly selected the performance in the zone where the rat was released at the starting position farthest from the hidden platform was assessed. One day after place navigation tests, the hidden platform was removed. The rats were placed randomly in a determined start point to assess spatial memory. Each rat swam for 120 s. Swimming distance and trace in quadrant D and time spent in quadrant D were recorded to assess the cognitive ability of rats. The swimming speed was also recorded to find out whether the differences between escape latency and swimming distance resulted from different swimming abilities.

### Hematoxylin-eosin (HE) staining

The rats were anesthetized with chloral hydrate (500 mg/kg, i.p.) at 12 d post-seizure and perfused via the left cardiac ventricle with 4% paraformaldehyde in phosphate buffered saline (PBS). The brain was removed and placed in the same fixative at 4°C for at least 24 h. The cerebellum and olfactory bulbs were removed. The first 6 mm of the frontal cerebral hemispheres was removed, and the next 8 mm, which contained the hippocampus, was collected and fixed in paraffin. The brains were placed in a 20% sucrose solution in phosphate buffer at room temperature for 48 h and in a 30% sucrose solution for at least 48 h. The brains were positioned on a freezing microtome stage, cut to a nominal thickness of 10 μm in the horizontal direction, followed by preserving at −20°C. HE staining was performed later. Surviving cells were defined as those with nuclei that were round shaped, with an intact cytoplasmic membrane and no nuclear condensation or distortion. Live pyramidal cells distributed in the area of hippocampal CA3 were observed using high magnification (×400).

### Terminal deoxynucleotidyl transferase dUTP nick-end labeling (TUNEL) assay

The One Step TUNEL Apoptosis Assay Kit (Beyotime Institute of Biotechnology, China) was used to detect the neuronal apoptosis. Continuous brain sections were dewaxed, hydrated, and incubated with proteinase K solution (20 μg/mL, without DNase) for 25 min at 37°C. Then, the sections were washed in PBS three times, 5 min each time. Next, DNA fragments were labeled by incubation with 50 μl TUNEL detection solution for 1 h at 37°C in a humidified chamber. The reaction was terminated by 2 × SSC termination solvent provided in the kit. After washed in PBS three times, the sections were mounted with anti-fluorescence quenching agent and examined under the fluorescence microscope.

### Immunohistochemistry

The 10-μm thick coronal sections were incubated with 3% H_2_O_2_ for 5 min to inhibit endogenous peroxidase activity and then rinsed in PBS for 5 min, followed by incubation with primary anti-glial fibrillary acidic protein (GFAP) antibody (Abcam, USA) diluted by 1:200 (glial fibrillary acidic protein) at 4°C overnight. Following incubation with species-specific biotinylated secondary antibody, the sections were incubated with peroxidase-labeled streptavidin, and the color was developed with diaminobenzene (Brusco et al., 1997). Finally, the sections were counterstained with hematoxylin. Negative controls were processed simultaneously by omitting the primary antibodies.

### Western blot

The dissected hippocampus tissues were washed with cold PBS, lysed in SDS lysis buffer and separated by SDS-PAGE. Then, the proteins were transferred to polyvinylidene difluoride membrane and immunoblotted with anti-GFAP primary antibody and horseradish peroxidase-conjugated secondary antibodies (all from Cell Signaling Technology, USA). Finally, detection was performed using the enhanced chemiluminescence reagents (Plus-ECL, PerkinElmer, MA, USA). β-actin was used as a control. The ImageJ software was used for the quantification of the bands.

### Statistical analysis

Statistical analyses were performed using SPSS version 19.0 (SPSS Inc., Chicago). The data were presented as the mean ± standard deviation (SD). Differences between two groups were determined using the *t*-Test. Differences among more than two groups were evaluated by one-way analysis of variance (ANOVA) followed by a Newman–Keuls *post-hoc* test. Chi-square test was applied to analyze the amount of subjects in stage 4 and 5 seizures. Differences were considered statistically significant at a *P* < 0.05.

## Results

### FK506 ameliorated the course of pilocarpine-induced epilepsy

Behavioral episodes induced by pilocarpine injections showed typical increases in their intensity and duration, gradually progressing toward SE. Compared with the pilocarpine-treated group, the FK506-treated group exhibited a longer latency period and a smaller percentage at stage V (Table 1). Control animals did not exhibit any behavioral seizure activity.

**Table 1:** Behavioral episodes of lithium-pilocarpine-induced status epilepticus rats after FK506 treatment.

**Groups**	**Numbers**	**The latency period to reaching stage IV–V ($\bar{x}$± s)**	**The percentage of animals reaching stage IV (%)**	**The percentage of animals reaching stage V (%)**
Pilocarpine group	54	47.4 ± 9.1	42.6	57.4
FK506 group	54	67.5 ± 9.7^*^	74.1^*^	25.9^*^

*The data are the mean ± SD*.

* *p < 0.05 vs. pilocarpine group*.

### FK506 ameliorated the neuronal loss in the hippocampus after SE

**Figure 1:**
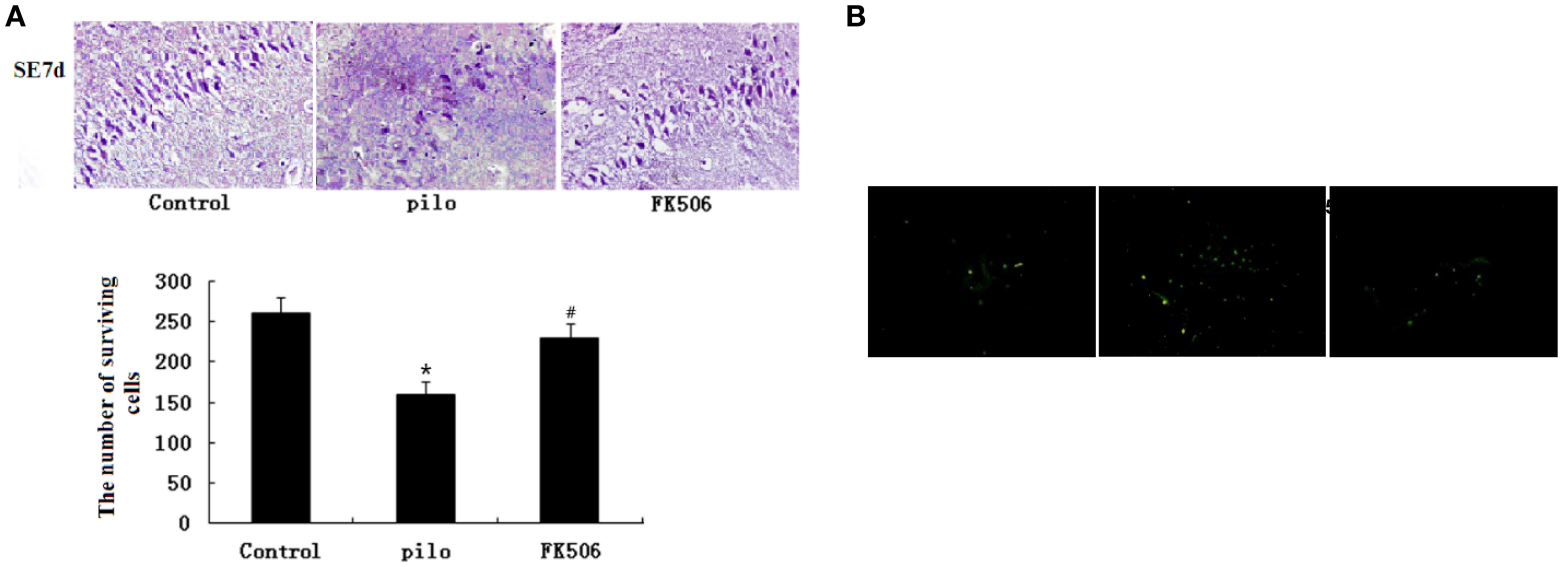
FK506 ameliorated the neuronal loss in the hippocampus of the lithium-pilocarpine-induced status epilepticus (SE) rats. **(A)** Hematoxylin-eosin (HE) staining of hippocampal CA3 pyramidal neurons 12 d after SE; Error bars represent mean ± SD. ^*^p < 0.05 vs. control, ^#^*p* < 0.05 vs. pilocarpine (pilo); one-way ANOVA. **(B)** TUNEL staining of hippocampal CA3 pyramidal neurons 12 d after SE. All experiments were performed in triplicate (*n* = 3). Photographs were taken at 40× magnification.

HE staining was used to detect neuronal changes in the hippocampus. The results showed that dead neurons with pyknotic nuclei were mainly located at hippocampal CA3 zone. Different from dead neurons, the live cells have round and palely stained nuclei. Compared to the control group, the pilocarpine-treated group had fewer surviving cells. However, when FK506 was given before pilocarpine injection, more neurons survived after SE (Figure 1A). The results from TUNEL staining also showed that the number of apoptotic neurons in pilocarpine-treated group was higher than that in control group. FK506 treatment could decrease the number of apoptotic neurons after seizures (Figure 1B).

### FK506 improved the learning and memory ability after SE

**Figure 2:**
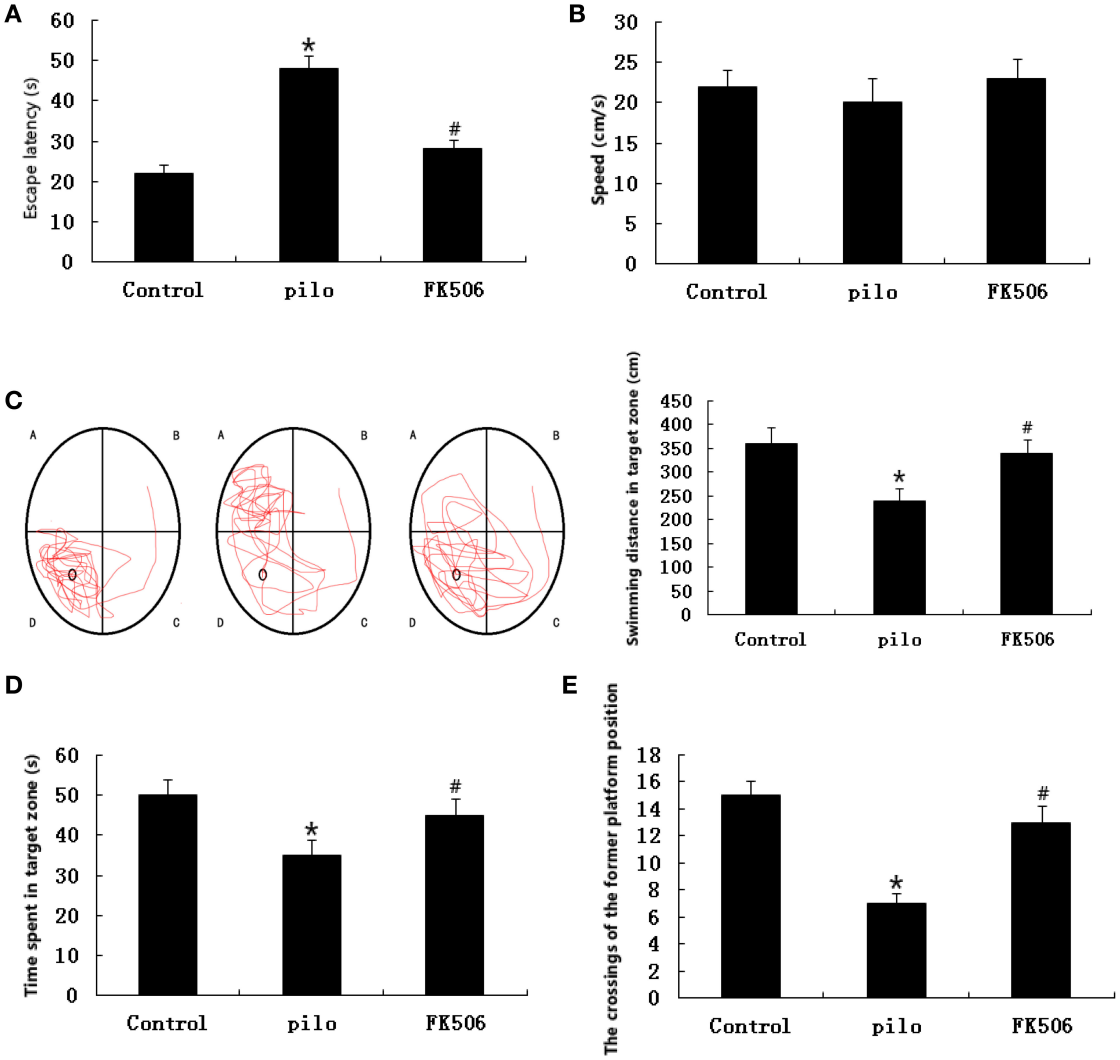
FK506 improved the learning and memory ability of the lithium-pilocarpine-induced status epilepticus (SE) rats. **(A)** Escape latency in the place navigation test; **(B)** Swimming speed in the spatial probe test; **(C)** Representative images of the path that the mice swam along to find the platform and swimming distance in the target zone in the spatial probe test; **(D)** Swimming time spent in the target zone in the spatial probe test; **(E)** Crossings to the former platform position in the spatial probe test. Error bars represent mean ± SD. ^*^p < 0.01 vs. control, ^#^*p* < 0.01 vs. pilocarpine (pilo); one-way ANOVA.

As shown by the results obtained in the Morris water maze test, the FK506 treatment improved the learning and memory ability of the rats. In the place navigation tests, the escape latency was significantly prolonged in the pilocarpine-treated group compared to the control group. In contrast, the prolonged latency was significantly reduced in the FK506-treated group (Figure 2A). As shown in Figure 2B, in the spatial probe tests, there were no significant differences in the swimming speeds of the rats between the indicated groups. Compared with the control group, the swimming distance in target zone was significantly decreased in the pilocarpine-treated group, while the swimming distance of the FK506-treated rats in the target zone was longer than that of the pilocarpine-treated rats (Figure 2C). Consistently, the rats in the FK506-treated group spent more time in the target zone and made more crossings to the position of the former platform than pilocarpine-treated group (Figures 2D,E).

### FK506 attenuated the GFAP expression and reactive astrogliosis after SE

**Figure 3:**
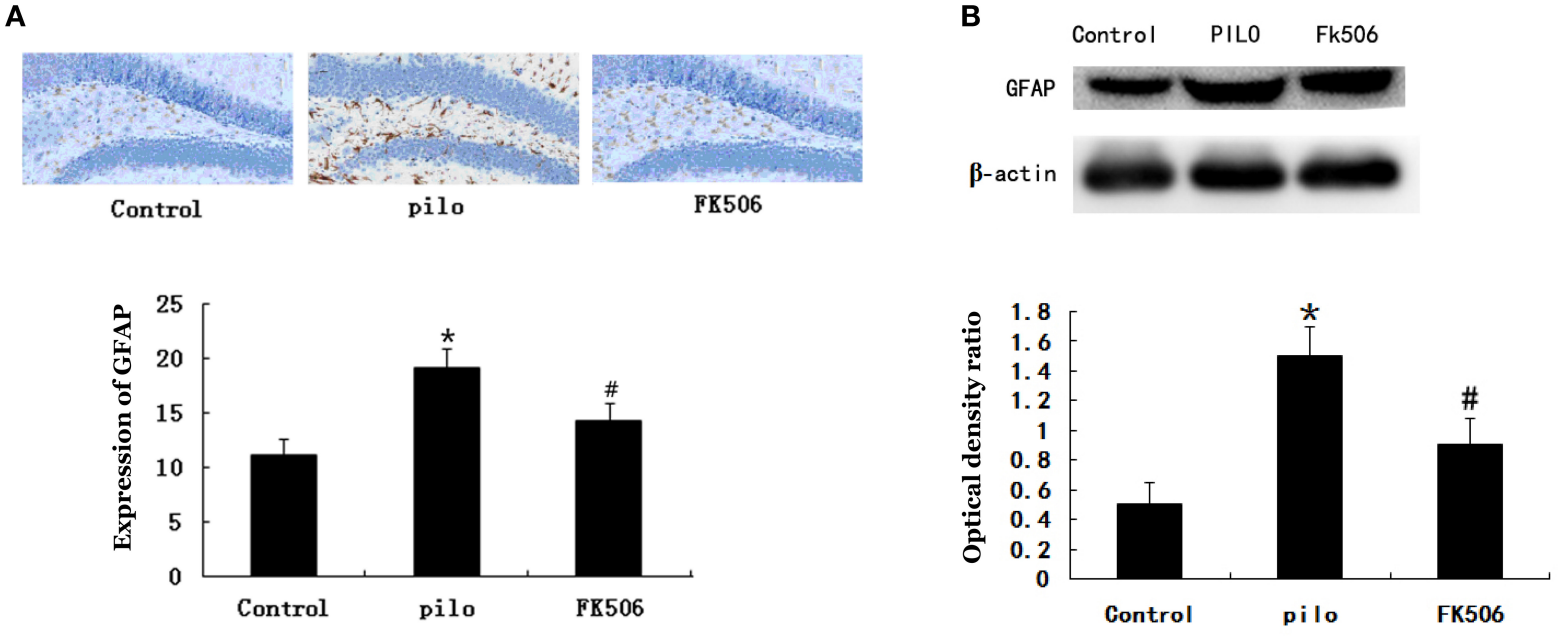
FK506 reduced the Glial fibrillary acidic protein (GFAP) expression in the hippocampus of the lithium-pilocarpine-induced status epilepticus (SE) rats. **(A)** Immunohistochemistry of GFAP using the GFAP monoclonal antibody (1:200). Photographs were taken at 40× magnification. **(B)** Western blot analysis of GFAP level. The relative density of GFAP was normalized to β-actin. All experiments were performed in triplicate (*n* = 3). Error bars represent mean ± SD. ^*^p < 0.01 vs. control, ^#^*p* < 0.01 vs. pilocarpine (pilo); one-way ANOVA.

GFAP, a cytoskeletal protein present in astroglial somata and projections, is used as a specific immunomarker of astrocytes. The immunohistochemistry analysis revealed that GFAP was expressed in cells 72 h after SE and that the number of GFAP-labeled cells increased 7 d after SE. As shown in Figure 3A, the GFAP expression was significantly increased in the pilocarpine-treated group compared with the control group, while the GFAP expression significantly decreased in the FK506-treated group when compared with the pilocarpine-treated group. Consistent with immunostaining data, the results from western blot showed that FK506 remarkably inhibited the increase of GFAP protein expression level after SE (Figure 3B). These results suggested that FK506 could significantly attenuate SE-induced reactive astrogliosis.

## Discussion

So far the pathogenesis and treatment of SE still need to be studied more. In this study, the results showed that FK506 treatment could ameliorate the course of pilocarpine-induced epilepsy, ameliorate the neuronal loss in the hippocampus and improved learning and memory ability. Furthermore, the FK506 treatment attenuated the reactive astrogliosis in the hippocampus after SE. These data suggest a protective role of FK506 in cognitive impairment after SE and this protective effect may be achieved by inhibiting the reactive astrogliosis.

Many factors contribute to epileptogenesis. Recently, prof. Heinemann and his colleagues proposed a “cholinergic hypothesis of temporal lobe epilepsy (TLE)” (Friedman et al., 2007). They thought that cholinergic innervation played a key role in the normal control of neuronal excitability and in higher cognitive processes (Friedman et al., 2007). The changes in the expression of key cholinergic proteins and the associated cholinergic dysfunction are key factors in the basic mechanisms underlying TLE (Friedman et al., 2007). Here, we used the muscarinic agonist pilocarpine to develop a rat model of status epileptic. Status epileptic is characterized by considerable cell loss in some regions of the brain. Cognitive impairment in epilepsy has been attributed to neuronal loss, glial growth, and hippocampal circuit dysfunction at higher seizure frequencies (Jung et al., 2006). This study showed that the pilocarpine-treated rats had more apoptotic neurons than the normal control group and the neuron loss was mainly located in the hippocampal CA3 zone. Meanwhile, the results obtained in the Morris water maze test demonstrated that the learning and memory ability of the pilocarpine-treated rats was reduced when compared with that of the control rats. All these results suggested that SE induced cognitive impairment.

Besides the cholinergic hypothesis, Recent findings from several laboratories suggested an astrocytic basis for epilepsy (Seifert et al., 2010). Astrogliosis is a pathological hallmark of an epileptic brain. It may contribute to seizure generation in epilepsy (Binder and Steinhäuser, 2006; Haydon and Carmignoto, 2006) and subsequent recurrent spontaneous seizures (Miller, 2005). Researchers demonstrated that astrocytes could impair the homeostatic control of network excitability and abnormal discharge synchronization in epilepsy. Multiple underlying mechanisms are proposed, such as impaired spatial K+ buffering (Hinterkeuser et al., 2000), dysfunctional glutamate homeostasis (Eid et al., 2008), dysfunctional gap junctions (Rouach et al., 2008), and changes in calcium buffering and gliotransmitter release (Pascual et al., 2005). Thus, under epileptic conditions, astrocytes do not seem to be neuro-supportive (de Lanerolle et al., 2010). Instead, seizures-induced activation of astrocytes could release pro-inflammatory cytokines and free radicals, both of which accelerate neuron loss (de Lanerolle et al., 2010). In addition, the loss of neurons, growth of glial, and hippocampal circuit dysfunction lead to the impairment of cognition in epilepsy (Byeon et al., 2015; Gant et al., 2015). So it suggests the possibility of a role of astrocyte activation played in epileptogenesis and cognitive impairment in epilepsy. In the present study, the expression level of GFAP, an indicator of astrocytes, started to rise at 72 h after SE and increased remarkably at 7 days after SE. Meanwhile, the GFAP expression was significantly increased in the pilocarpine-treated group compared with the control group. These findings suggest that SE induced reactive astrogliosis, which was consistent with the previous studies (Damaye et al., 2011; Hong et al., 2012; Li et al., 2012; Sherafat et al., 2013). However, further studies are required to explore the exact role of astrocyte activation in cognitive impairment associated with SE.

As muscarinic receptor activation could increase intracellular Ca^2+^ (Egorov and Müller, 1999), the calcineurin inhibitor FK506 may be useful in the treatment of pilocarpine-induced epilepsy. Many studies suggested a role of FK506 in neuroprotection. In a rat model of middle cerebral artery occlusion, an intravenous injection of FK506 1 min after vascular occlusion reduced cortical lesions by 60–70% (Nito et al., 2011). In addition, FK506 exerted a neuroprotective effect in ischemia (Ricci et al., 2009; Gant et al., 2015; Malvezzi and Rostaing, 2015; Peixoto-Santos et al., 2015; Zou et al., 2015). FK506 protected hippocampal neurons from forebrain ischemia in the Mongolian gerbil (Nito et al., 2011). FK506 also prevented mossy fibers generation in the hippocampus and stimulation-induced epileptogenesis (Gant et al., 2015). In the present study, we observed that FK506 increased the latency period to seizures and decreased the maximal intensity of seizures. FK506 treatment could also greatly decrease the number of apoptotic neurons after seizures. In addition, FK506 reduced the escape latency and prolong the swimming distance in the Morris water maze test. Thus, we concluded that FK506 could improve impaired cognition after pilocarpine-induced SE in rats. On the other hand, we found that the FK506 treatment lessened the activation of astrocyte in the rats' hippocampus after SE. In addition, FK506 treatment could markedly decrease the GFAP level, suggesting that FK506 attenuated reactive astrogliosis, which is in agreement with previous reports (Zawadzka and Kaminska, 2005; Yoshiyama et al., 2007). As FK506 is an immunosuppression, the risk of FK506 should be assessed in the further research.

In summary, the present study suggested that treatment with FK506 might improve cognitive impairment by preventing astrocyte activation after SE, suggesting that FK506 may be a promising agent for treatment of SE-induced cognitive impairment.

## Author contributions

JL: performed the study, wrote the manuscript, and participated in designing of the study; ZS: performed the study and participated in writing the manuscript; SL: helped to perform the study and collected the data; ZH: collected the data and analyzed the data; YH and TZ: helped to perform the study; AW: conceived and designed the research; All authors read and approved the final manuscript.

### Conflict of interest statement

The authors declare that the research was conducted in the absence of any commercial or financial relationships that could be construed as a potential conflict of interest.

## References

[B1] Agarwal N. K. Mediratta P. K. Sharma K. K. (2011). Effect of lamotrigine, oxcarbazepine and topiramate on cognitive functions and oxidative stress in PTZ-kindled mice. Seizure 20, 257–262. 10.1016/j.seizure.2010.12.006 21247777

[B2] Binder D. K. Steinhäuser C. (2006). Functional changes in astroglial cells in epilepsy. Glia 54, 358–368. 10.1002/glia.20394 16886201

[B3] Brusco A. Saavedra J. P. García G. Tagliaferro P. Evangelista de Duffard A. M. Duffard R. (1997). 2,4-dichlorophenoxyacetic acid through lactation induces astrogliosis in rat brain. Mol. Chem. Neuropathol. 30, 175–185. 10.1007/BF02815096 9165484

[B4] Byeon J. H. Kim G. H. Kim J. Y. Sun W. Kim H. Eun B. L.. (2015). Cognitive dysfunction and hippocampal damage induced by hypoxic-ischemic brain injury and prolonged febrile convulsions in immature rats. J. Korean Neurosurg. Soc. 58, 22–29. 10.3340/jkns.2015.58.1.22 26279809 PMC4534735

[B5] Damaye C. A. Wu L. Peng J. He F. Zhang C. Lan Y.. (2011). An experimental study on dynamic morphological changes and expression pattern of GFAP and synapsin i in the hippocampus of MTLE models for immature rats. Int. J. Neurosci. 121, 575–588. 10.3109/00207454.2011.598979 21812737

[B6] de Lanerolle N. C. Lee T. S. Spencer D. D. (2010). Astrocytes and epilepsy. Neurotherapeutics 7, 424–438. 10.1016/j.nurt.2010.08.002 20880506 PMC5084304

[B7] Egorov A. V. Müller W. (1999). Subcellular muscarinic enhancement of excitability and Ca2+-signals in CA1-dendrites in rat hippocampal slice. Neurosci. Lett. 261, 77–80. 10.1016/S0304-3940(99)00007-5 10081931

[B8] Eid T. Williamson A. Lee T. S. Petroff O. A. de Lanerolle N. C. (2008). Glutamate and astrocytes–key players in human mesial temporal lobe epilepsy? Epilepsia 49(Suppl. 2), 42–52. 10.1111/j.1528-1167.2008.01492.x 18226171

[B9] Elsharkawy A. E. Alabbasi A. H. Pannek H. Oppel F. Schulz R. Hoppe M. Hamad A. P.. (2009). Long-term outcome after temporal lobe epilepsy surgery in 434 consecutive adult patients. J. Neurosurg. 110, 1135–1146. 10.3171/2008.6.JNS17613 19025359

[B10] Friedman A. Behrens C. J. Heinemann U. (2007). Cholinergic dysfunction in temporal lobe epilepsy. Epilepsia 48(Suppl. 5), 126–130. 10.1111/j.1528-1167.2007.01300.x 17910592

[B11] Gant J. C. Chen K. C. Kadish I. Blalock E. M. Thibault O. Porter N. M.. (2015). Reversal of aging-related neuronal Ca^2+^ dysregulation and cognitive impairment by delivery of a transgene encoding FK506-binding protein 12.6/1b to the Hippocampus. J. Neurosci. 35, 10878–10887. 10.1523/JNEUROSCI.1248-15.2015 26224869 PMC4518058

[B12] Haydon P. G. Carmignoto G. (2006). Astrocyte control of synaptic transmission and neurovascular coupling. Physiol. Rev. 86, 1009–1031. 10.1152/physrev.00049.2005 16816144

[B13] Hinterkeuser S. Schröder W. Hager G. Seifert G. Blümcke I. Elger C. E.. (2000). Astrocytes in the hippocampus of patients with temporal lobe epilepsy display changes in potassium conductances. Eur. J. Neurosci. 12, 2087–2096. 10.1046/j.1460-9568.2000.00104.x 10886348

[B14] Hong S. Xin Y. HaiQin W. GuiLian Z. Ru Z. ShuQin Z.. (2012). The PPARgamma agonist rosiglitazone prevents cognitive impairment by inhibiting astrocyte activation and oxidative stress following pilocarpine-induced status epilepticus. Neurol. Sci. 33, 559–566. 10.1007/s10072-011-0774-2 21915647

[B15] Jansson L. Wennström M. Johanson A. Tingström A. (2009). Glial cell activation in response to electroconvulsive seizures. Prog. Neuropsychopharmacol. Biol. Psychiatry 33, 1119–1128. 10.1016/j.pnpbp.2009.06.007 19540297

[B16] Jung K. H. Chu K. Lee S. T. Kim J. Sinn D. I. Kim J. M.. (2006). Cyclooxygenase-2 inhibitor, celecoxib, inhibits the altered hippocampal neurogenesis with attenuation of spontaneous recurrent seizures following pilocarpine-induced status epilepticus. Neurobiol. Dis. 23, 237–246. 10.1016/j.nbd.2006.02.016 16806953

[B17] Kaminska B. Gaweda-Walerych K. Zawadzka M. (2004). Molecular mechanisms of neuroprotective action of immunosuppressants–facts and hypotheses. J. Cell. Mol. Med. 8, 45–58. 10.1111/j.1582-4934.2004.tb00259.x 15090260 PMC6740149

[B18] Li D. Li P. He Z. Cen D. Meng Z. Liang L.. (2012). Human intravenous immunoglobulins suppress seizure activities and inhibit the activation of GFAP-positive astrocytes in the hippocampus of picrotoxin-kindled rats. Int. J. Neurosci. 122, 200–208. 10.3109/00207454.2011.639470 22082354

[B19] Malvezzi P. Rostaing L. (2015). The safety of calcineurin inhibitors for kidney-transplant patients. Expert Opin. Drug Saf. 14, 1531–1546. 10.1517/14740338.2015.1083974 26329325

[B20] Miller G. (2005). Neuroscience. The dark side of glia. Science 308, 778–781. 10.1126/science.308.5723.778 15879185

[B21] Motamedi G. Meador K. (2003). Epilepsy and cognition. Epilepsy Behav. 4(Suppl. 2), S25–S38. 10.1016/j.yebeh.2003.07.004 14527481

[B22] Nito C. Ueda M. Inaba T. Katsura K. Katayama Y. (2011). FK506 ameliorates oxidative damage and protects rat brain following transient focal cerebral ischemia. Neurol. Res. 33, 881–889. 10.1179/1743132811Y.0000000019 22004713

[B23] Pascual O. Casper K. B. Kubera C. Zhang J. Revilla-Sanchez R. Sul J. Y.. (2005). Astrocytic purinergic signaling coordinates synaptic networks. Science 310, 113–116. 10.1126/science.1116916 16210541

[B24] Peixoto-Santos J. E. Velasco T. R. Galvis-Alonso O. Y. Araujo D. Kandratavicius L. Assirati J. A.. (2015). Temporal lobe epilepsy patients with severe hippocampal neuron loss but normal hippocampal volume: extracellular matrix molecules are important for the maintenance of hippocampal volume. Epilepsia 56, 1562–1570. 10.1111/epi.13082 26218733

[B25] Pestana R. R. Kinjo E. R. Hernandes M. S. Britto L. R. (2010). Reactive oxygen species generated by NADPH oxidase are involved in neurodegeneration in the pilocarpine model of temporal lobe epilepsy. Neurosci. Lett. 484, 187–191. 10.1016/j.neulet.2010.08.049 20732386

[B26] Pouzet B. Zhang W. N. Feldon J. Rawlins J. N. (2002). Hippocampal lesioned rats are able to learn a spatial position using non-spatial strategies. Behav. Brain Res. 133, 279–291. 10.1016/S0166-4328(02)00007-4 12110461

[B27] Racine R. J. (1972). Modification of seizure activity by electrical stimulation. II. Motor seizure. Electroencephalogr. Clin. Neurophysiol. 32, 281–294. 10.1016/0013-4694(72)90177-0 4110397

[B28] Ricci G. Volpi L. Pasquali L. Petrozzi L. Siciliano G. (2009). Astrocyte-neuron interactions in neurological disorders. J. Biol. Phys. 35, 317–336. 10.1007/s10867-009-9157-9 19669420 PMC2750745

[B29] Rodriguez-Alvarez N. Jimenez-Mateos E. M. Dunleavy M. Waddington J. L. Boylan G. B. Henshall D. C.. (2015). Effects of hypoxia-induced neonatal seizures on acute hippocampal injury and later-life seizure susceptibility and anxiety-related behavior in mice. Neurobiol. Dis. 83, 100–114. 10.1016/j.nbd.2015.08.023 26341542

[B30] Rouach N. Koulakoff A. Abudara V. Willecke K. Giaume C. (2008). Astroglial metabolic networks sustain hippocampal synaptic transmission. Science 322, 1551–1555. 10.1126/science.1164022 19056987

[B31] Seifert G. Carmignoto G. Steinhäuser C. (2010). Astrocyte dysfunction in epilepsy. Brain Res. Rev. 63, 212–221. 10.1016/j.brainresrev.2009.10.004 19883685

[B32] Sherafat M. A. Ronaghi A. Ahmad-Molaei L. Nejadhoseynian M. Ghasemi R. Hosseini A.. (2013). Kindling-induced learning deficiency and possible cellular and molecular involved mechanisms. Neurol. Sci. 34, 883–890. 10.1007/s10072-012-1142-6 22744648

[B33] Swann J. W. (2004). The effects of seizures on the connectivity and circuitry of the developing brain. Ment. Retard. Dev. Disabil. Res. Rev. 10, 96–100. 10.1002/mrdd.20018 15362163

[B34] Vervliet T. Parys J. B. Bultynck G. (2015). Bcl-2 and FKBP12 bind to IP3 and ryanodine receptors at overlapping sites: the complexity of protein-protein interactions for channel regulation. Biochem. Soc. Trans. 43, 396–404. 10.1042/BST20140298 26009182

[B35] Wang J. Guo R. Liu S. Chen Q. Zuo S. Yang M.. (2014). Molecular mechanisms of FK506-induced hypertension in solid organ transplantation patients. Chin. Med. J. 127, 3645–3650. 10.3760/cma.j.issn.0366-6999.20141176 25316243

[B36] Yoshiyama Y. Higuchi M. Zhang B. Huang S. M. Iwata N. Saido T. C.. (2007). Synapse loss and microglial activation precede tangles in a P301S tauopathy mouse model. Neuron 53, 337–351. 10.1016/j.neuron.2007.01.010 17270732

[B37] Zawadzka M. Kaminska B. (2005). A novel mechanism of FK506-mediated neuroprotection: downregulation of cytokine expression in glial cells. Glia 49, 36–51. 10.1002/glia.20092 15390105

[B38] Zou X. F. Song B. Duan J. H. Hu Z. D. Cui Z. L. Gu C. (2015). Prolonged ischemia elicits acute allograft rejection involved in CXCR3 activation in rat kidney transplants. Transpl. Immunol. 33, 103–109. 10.1016/j.trim.2015.08.001 26303820

